# Genomic Analysis Reveals the Role of New Genes in Venom Regulatory Network of Parasitoid Wasps

**DOI:** 10.3390/insects16050502

**Published:** 2025-05-07

**Authors:** Bo Zhang, Yifan Bu, Jiqiang Song, Bo Yuan, Shan Xiao, Fang Wang, Qi Fang, Gongyin Ye, Yi Yang, Xinhai Ye

**Affiliations:** 1 State Key Laboratory of Rice Biology and Breeding, Zhejiang University, Hangzhou 310058, China; zhangbo2021@zju.edu.cn (B.Z.); buyifan@zju.edu.cn (Y.B.);; 2 Ministry of Agricultural and Rural Affairs Key Laboratory of Molecular Biology of Crop Pathogens and Insects, Zhejiang University, Hangzhou 310058, China; 3 College of Advanced Agriculture Science, Zhejiang A&F University, Hangzhou 311300, China; 4 Zhejiang Key Laboratory of Biology and Ecological Regulation of Crop Pathogens and Insects, Zhejiang A&F University, Hangzhou 311300, China

**Keywords:** parasitoid wasp, new gene, venom, evolution, gene regulatory network

## Abstract

New genes play a critical role in phenotypic diversity and evolutionary innovation. Parasitoid wasps, a highly abundant and diverse group of insects, parasitize other arthropods and exhibit remarkable evolutionary adaptations, such as evading host immune responses and exploiting host resources. However, the specific contributions of new genes to their unique traits remain poorly understood. Here, we identified 480 new genes that emerged after the *Nasonia-Pteromalus* divergence. Among these, 272 (56.7%) originated through DNA-mediated duplication, representing the largest proportion, followed by 77 (16.0%) derived from RNA-mediated duplication and 131 (27.3%) that arose de novo. Comparative analysis revealed that these new genes generally have shorter coding sequences and fewer exons compared to single-copy older genes conserved in the seven parasitoid wasps. These new genes are predominantly expressed in the reproductive glands and exhibit venom gland-biased expression. Notably, gene co-expression network analysis further identified that a new gene may act as a hub by interacting with older genes to regulate venom-related networks rather than directly encoding venom proteins. Together, our findings provide novel insights into the role of new genes in driving venom innovation in parasitoid wasps.

## Simple Summary

Parasitoid wasps are insects that lay eggs on or inside other arthropods, evolving extraordinary abilities to avoid their hosts’ defenses and use their resources. While the new genes are thought to drive adaptive evolution, their exact roles remain unknown. This study identified a set of new genes that emerged during the evolution of Pteromalidae wasps. Most of these new genes formed by gene duplication, while others emerged de novo birth. These genes are shorter, simpler in structure, and primarily enriched in reproductive organs and venom glands. A key finding revealed that one new gene acts as a central hub, coordinating with older genes to regulate venom-related gene network instead of making venom proteins directly. This shows how new genes collaborate with existing ones to create evolutionary innovations in parasitoid wasps.

## 1. Introduction

The emergence of new genes has been proposed as a key driver of phenotypic diversity and innovation across the tree of life [1,2]. New genes arise in specific genomic loci within a species at a particular point in evolutionary time where they did not previously exist [3]. The birth of new genes occurs through diverse molecular mechanisms, encompassing gene duplication, transposable element protein domestication, lateral gene transfer, gene fusion, and de novo origination [4]. These diverse mechanisms have facilitated widespread new gene origination across taxa, from plants to animals [5,6,7]. In primates, the brain and testis serve as evolutionary hotspots for the recruitment of new genes, likely contributing to the development of unique traits, biological functions, and behaviors [8,9]. The birth of organ-specific new genes significantly enhances our understanding of the frequency with which these new genes contribute to phenotypic evolution.

The vast species diversity and evolutionary complexity of insects provide a critical framework for investigating genetic novelty and the role of new genes in adaptation. A well-known new gene, *Jingwei*, which was the first reported new gene in *Drosophila*, arising through retrotransposition and neofunctionalization. *Jingwei* acquired a novel expression pattern specifically in the testis [10]. Since this discovery, numerous studies have demonstrated that new genes can influence male courtship behaviors or fertility-related functions over various evolutionary timescales in *Drosophila*. Examples include *sphinx*, *nsr*, *Umbrea*, *saturn*, and *atlas* [11,12,13,14]. In addition, *Lushu*, as a new gene identified in *Plutella xylostella,* also exhibited the male-biased expression pattern, which potentially results in enhancing sperm competition [15]. While most new genes show prominent roles in male reproductive organs, some exceptions have been observed. For instance, a new duplicate gene of parasitoid wasp *Nasonia vitripennis* (*VenomY*) affected detoxification and immunity genes in envenomated fly hosts [16]. These findings suggest that new genes can act as reservoirs of genetic innovation, contributing to diverse biological functions and driving organ-specific adaptations.

Parasitoid wasps represent a highly diverse and abundant group of insects that obligately parasitize other arthropods [17,18]. Among the diverse biological characteristics of parasitoid wasps, venom is particularly hypothesized to represent key evolutionary innovations that have facilitated the ecological success of parasitoid wasps. Recent studies have revealed venom genes in parasitoid wasp have exhibited a high turnover rate, primarily driven by the co-option of other pre-existing genes [19,20]. However, systematic research on the role of new genes in the origination and evolution of venom in parasitoid wasps remains limited.

In this study, we performed whole-genome syntenic alignments and identified 480 new genes in the parasitoid wasp *Pteromalus puparum*, which diverged after *Nasonia-Pteromalus* lineage split approximately 8 million years ago (MYA). Our findings reveal that the evolutionary patterns of new genes in parasitoid wasps, including origination mechanisms and tissue-specific expression, are comparable to those observed in *Drosophila* and mammals. Notably, these new genes exhibit tissue-specific expression, being predominantly expressed in reproductive glands and displaying venom-biased expression profiles. Although the new genes did not directly exhibit venom-related functions, they acted as hub genes within venom-related gene networks by interacting with older genes. These findings suggest that new genes may play a pivotal role in driving the innovative evolution of venom in parasitoid wasps.

## 2. Materials and Methods

### 2.1. Data Collection

We collected seven high-quality genomes of parasitoid wasps in the family Pteromalidae, including *P. puparum*, *P. venustus*, *P. qinghaiensi*, *N. vitripennis*, *A. calandrae*, *P. vindemmiae*, and *T. elegans* (Supplementary Table S1). The completeness of the genome assemblies was assessed using the Benchmarking Universal Single-Copy Orthologs (BUSCO v5.7.1) with the insecta_odb10 database [21]. The protein-coding sequences were downloaded from the corresponding data sources, and the longest transcript was considered representative of the gene with annotations for multiple isoforms.

### 2.2. Phylogenetic Analysis

To construct the phylogenetic relationships, we utilized protein sequences from seven species and clustered them into orthologous groups using OrthoFinder v2.2.7 [22]. The 3798 one-to-one orthogroups (also referred to as single-copy genes) shared among the seven parasitoid wasp species were selected for constructing the phylogenetic tree. Protein sequences were aligned and filtered using MAFFT v7 and trimAl v1.2 with default parameters [23,24]. The alignments for each orthogroup were then concatenated to generate a supergene, which was utilized for subsequent tree construction. The phylogenetic tree was constructed using maximum likelihood (ML) with IQ-TREE v2.1.2, employing the best-fit model (JTT + F + I + R9) estimated by ModelFinder [25,26]. Statistical support for the phylogenetic tree was evaluated using Ultrafast bootstrap analysis with 1000 replicates. Divergence times were estimated using r8s v1.81 [27], with calibration time points based on previous research [28].

### 2.3. Dating the Protein-Coding Genes Within Parasitoid Wasps

We utilized a whole-genome synteny-based pipeline (SBP) to identify new genes [6], selecting *P. puparum* as the reference species and generating alignments with the other six species using LASTZ v1.04.03 (https://lastz.github.io/lastz/ accessed on 4 March 2025). The genes were assigned branch numbers ranging from 0 (indicating genes shared by all selected parasitoid wasps) to 6 (indicating genes specific to *P. puparum*) to denote the age of each gene, with higher branch numbers corresponding to younger genes. New genes were defined as those that originated after the *Nasonia-Pteromalus* split (~8 MYA) and lacked a corresponding syntenic locus in *N. vitripennis*, *A. calandrae*, *P. vindemmiae*, and *T. elegans*, following the stringent criterion outlined by previous study [6]. In detail, we excluded genes where more than 70% exonic regions overlapped with repetitive elements and removed the genes with patchy phylogenetic distribution. Finally, to validate the reliability of the new genes, we performed BLASTP alignments for the 480 new genes against the NCBI RefSeq Hymenoptera protein sequence database, using the following criteria: sequence identity > 20% and E-value < 10^−5^. The identified new genes were classified into three categories of origin mechanisms [6]: DNA-mediated duplication, RNA-mediated duplication (retrogenes), and de novo birth. Retrogenes were identified as intronless genes derived from parental genes with at least one intron; otherwise, they were classified as gene duplications. To enhance identification accuracy of de novo genes originating from ancestrally non-coding regions, we implemented an additional pipeline [5] to detect the stepwise origination process of ORFs with the following sequential criteria: (1) ORFs must lack homologs in *T. elegans* and possess no more than one homolog in each species; (2) ORFs must show no more than one homologous DNA sequence per species, with at least one ortholog demonstrating more than 20% sequence coverage. Subsequently, we validated the structural annotation and orthology relationship of candidate de novo genes using TOGA [29] with parameters (–cb 3,5 –cjn 500).

### 2.4. Selection Analysis

After performing all-against-all BLASTP v2.12, we made the inference of old duplicate paralogs assigned to branch 0–3 and parent–child duplicate relationship. To examine the selection pressure exerted on the new duplicate genes, we compared them with their closest parental paralogous genes to calculate the ratio of non-synonymous substitution rate (Ka) to synonymous substitution rate (Ks). According to gene age and alignment, we made the inference of old duplicate paralogs. Initially, we generated protein-coding sequence alignments between parental and child genes using MAFFT v7 based on the dating inference [23]. Subsequently, we used the PAL2NAL tool to convert the protein alignments into codon-level alignments [30]. The paralogous Ka/Ks tests were conducted utilizing the PAMLv4.9 package [31], and the likelihood ratio test (LRT) was employed to calculate the *p*-value, assuming Ka/Ks = 0.5. Genes with Ka > 0.5, Ks > 5, or Ks values exceeding 1.5 times the interquartile range of the Ks distribution were excluded. Duplicate genes with Ka/Ks < 0.5 and a *p*-value < 0.05 were considered to be under negative selection, indicating evolutionary constraints on both copies.

### 2.5. Expression Pattern of New Genes

We downloaded the RNA-Seq data of testis, ovary, venom gland, and salivary gland at 3.5 days and 5.5 days and gut data at 3.5 days and 5.5 days (Supplementary Table S1). We employed Fastp v0.23.4 to eliminate adapters and low-quality bases [32]. The filtered reads were mapped to the *P. puparum* genome using Bowtie2 v2.2.9, and the output was processed with RSEM v1.3.3 to generate transcript per million (TPM) values [33,34]. To analyze the expression patterns of the new genes, we compared them with one-to-one single-copy genes shared by the seven species (older genes) across various tissues. Genes with TPM > 1 in at least one tissue were classified as expressed. The tissue-specific index (*τ*) was calculated as follows:

$$\tau =\frac{\sum_{i=1}^{N}\left(1-Xi\right)}{N-1};Xi=\frac{Xi}{\underset{1\leq i\leq n}{\max}(Xi)}$$

where Xi is the gene expression level in tissue i, and *N* is the tissue numbers. Genes with *τ* > 0.85 were classified as tissue-specific expressed genes. To further quantify expression abundance and breadth across three gene-age groups (potential branch 0–1, branch 2–3, and branch 4–6), we calculated the maximum expression observed in the seven tissues and counted the number of tissues in which genes were expressed.

### 2.6. Co-Expression Network Analysis

We used the R package WGCNA v 1.72 to construct the weighted gene co-expression networks [35]. A total of 14,622 genes were used to build the co-expression network with the parameters as follows: network type = unsigned, soft power = 9, module identification method = dynamic tree cut, minimum module size = 30, and the threshold to merge modules with a high similarity = 0.5. Based on the expression patterns, we clustered all selected genes into 17 modules. Principal component analysis was performed on the genes within each module, and the value of the first principal component, termed module eigengene (ME), was used to represent the overall level of gene expression in the module. We treated tissues as traits and calculated the correlation between MEs and traits, along with the corresponding *p*-values, to identify key modules associated with specific traits. For each module, gene significance (GS) was defined as the absolute value of the correlation between the gene and the trait. The selected network regulated by the new genes was visualized using Cytoscape v3.9.1 [36]. Gene Ontology (GO) enrichment analysis was conducted using the R package clusterProfiler [37].

### 2.7. Total RNA Isolation, cDNA Synthesis, and Real-Time Quantitative PCR

Total RNA was isolated using RNAiso Plus (Takara Bio, Otsu, Japan). Then, the first-strand cDNA was prepared from 1.0 µg total RNA by reverse transcription using the TransScript One-Step gDNA Removal and cDNA Synthesis SuperMix Kit (TransGen Biotech, Beijing, China). The resulting PCR products were then sequenced. To quantify the expression of the new gene *Ppup071090.1* across various tissues of *P. puparum*, RT-qPCR was performed in testes, ovaries, venom glands, larval salivary glands, and carcasses (without ovaries and venom glands), and the primers for RT-qPCR are detailed in Supplementary Table S7. This was conducted using the Bio-Rad CFX 96 Real-Time Detection System (Bio-Rad, Hercules, CA, USA) with ChamQ SYBR Color qPCR Master Mix (Vazyme, Nanjing, China), following the manufacturer’s instructions. The relative expression levels were normalized to the reference gene 18s using the 2^−ΔΔCT^ method [38]. All quantitative data were expressed as the mean ± standard error of the mean (SEM) of three independent biological replicates. Statistical significance was performed using a one-way analysis of variance followed by Tukey’s honestly significant difference test for multiple comparisons. This statistical analysis was conducted on R software v4.4.2.

## 3. Results

### 3.1. Identification and Origin of New Genes in the Parasitoid Wasps

To identify and investigate the origin of new genes in parasitoid wasps, we screened the genomes of seven species across the Pteromalidae family, including *Pteromalus puparum*, *P. venustus*, *P. qinghaiensis*, *Nasonia vitripennis*, *Anisopteromalus calandrae*, *Pachycrepoideus vindemmiae*, and *Theocolax elegans*. *T. elegans* and *P. vindemmiae* were strategically chosen as outgroups to establish evolutionary context, with *N. vitripennis* and *A. calandrae* serving as closely related ingroup taxa. These seven genomes are of high quality, with average percentage of complete single-copy BUSCO genes of 97.3% (Supplementary Table S2). Due to the deep evolutionary timescales involved, tracing the processes of new gene formation is inherently challenging. However, focusing on closely related species can offer insights over shorter evolutionary timescales [39]. To this end, we first reconstructed a phylogenetic tree using 3798 single-copy genes identified across the seven species. Our analysis indicated that these parasitoid wasps shared close evolutionary relationships, with an estimated divergence time of approximately 80 million years (MY), and the most closely related species diverged around 1.49 MY ago. Thus, this group of selected Pteromalidae wasps represents an excellent model for systematically investigating the formation of new genes in parasitoid wasps.

**Figure 1:**
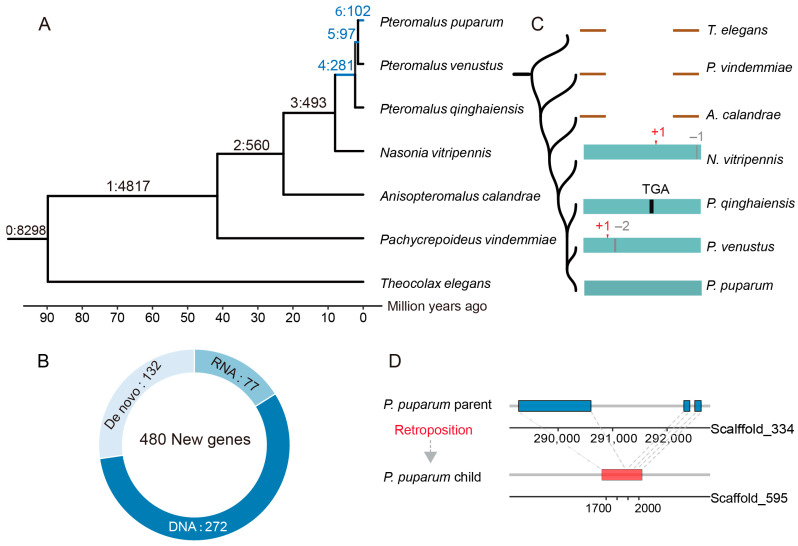
New genes identified in parasitoid wasps. (**A**) Distribution of new genes in the phylogenetic tree. Branches 0–6 were used as the distinct gene-age groups based on the synteny-based pipeline. Genes that emerged after *Nasonia-Pteromalus* split (branch 4–6) were identified as the new genes. Branches 4–6 (blue): Number of new genes. (**B**) Numbers and proportions of the new genes originated from DNA-mediated duplication, RNA-mediated duplication (retroposition), and de novo birth. (**C**) Stepwise origination process for an example of a de novo gene. The black bar represents a premature stop codon, the red arrow represents the frameshift insertion, and the grey bar indicates the frameshift deletion. Inserted bases are marked in red with their count shown. Deleted bases are marked in gray with their count shown. (**D**) The origination process for an example of retrotransposon. The blue or red box indicates exon.

To minimize any confusion arising from the frequent genomic rearrangements in Hymenoptera evolution and potential inconsistencies in gene annotation quality, we employed a whole-genome alignment-based approach to identify new genes in parasitoid wasps [6]. Using the LASTZ-MULTIZ pipeline [6,40], we aligned the genomes of the seven parasitoid wasp species. *P. puparum* was selected as the reference genome for its high-quality assembly, comprehensive gene annotations supported by diverse RNA sequencing datasets (e.g., IsoSeq, CAGE-Seq, and PAS-Seq), and its status as an emerging model organism with increasing functional studies [41,42,43]. For each of the 17,656 protein-coding genes in *P. puparum*, we assigned an origin age to corresponding phylogenetic tree branches based on ortholog presence or absence across the other species. Genes that originated after recent *Nasonia-Pteromalus* split (~8 MYA) were classified as “new genes”. In total, we identified 480 new genes that emerged during the evolution of Pteromalidae wasps, accounting for 2.71% of the entire gene set in *P. puparum* (Figure 1A). We observed a high rate of new gene birth in the Pteromalidae lineage, with 5.34 new genes emerging per MY (480/89.83 MY). Specifically, 102 genes emerged after the divergence of *P. puparum* and *P. venustus* around 1.49 MYA, corresponding to a birth rate of 68.46 new genes per MY. This rate is notably higher than those observed in *Drosophila* and other lineages [44], indicating that the rapid formation of new genes may play a crucial role in the adaptive evolution of parasitoid wasps.

We next classified the new genes into three categories based on their mechanisms of origin, including DNA-mediated duplication, RNA-mediated duplication, and de novo birth [6]. Over half of the new genes (56.67%, 272/480) originated through DNA-mediated duplication, following by RNA-mediated duplication (16.0%, 77/480) and de novo birth (27.29%, 131/480) (Figure 1B, Supplementary Table S3). This pattern was consistent with findings in other lineages, where most new genes arose through DNA duplication [7,44,45]. As another major mechanism of gene duplication, RNA-mediated duplication (retroposition) is a process where mRNAs are reverse-transcribed into DNAs and then insert back into a new position on the genome. A hallmark of RNA-mediated duplication is the transition from a multi-exon ancestral gene to a single-exon new gene. These duplicates retain sequences from their parent genes and contribute to phenotypic evolution through various mechanisms, including neofunctionalization, hypofunctionalization, subfunctionalization, and gene dosage regulation [46]. For example, a retrogene in *P. puparum* (~1.49 MYA), which is lineage-specific, is a single-exon gene derived from a three-exon parental gene (Figure 1D).

Recent studies have also emphasized the role of de novo genes in driving rapid protein diversity across various taxa, including *Drosophila* and mammals [8,47]. In our study, we identified several de novo genes during the evolution of parasitoid wasps. Unlike orphan genes (lineage-specific genes lacking homologous sequences in distantly related species), our de novo genes were traced to ancestral non-coding DNA sequences. This was facilitated by our dataset of closely related species, which allowed us to reconstruct the stepwise origination processes of de novo genes. By comparing novel open reading frames (ORFs) with their closest outgroup non-coding sequences, we identified key mutations, such as indels (insertions and deletions) and substitutions, that transformed non-coding DNA into coding sequences. For instance, *Ppup035000.1* (putative uncharacterized protein) represents a stepwise de novo gene formation process involving multiple frameshifts and substitutions. Orthologous non-coding sequences for *Ppup035000.1* were identified in both *N. vitripennis* and *P. venustus*, which exhibited frameshift mutations. Additionally, a premature stop codon was detected in closely related species, namely *P. qinghaiensis*, further illustrating this stepwise transformation (Figure 1C).

Overall, our genome-wide alignment-based approach yielded a high-confidence dataset of new genes involved in evolution of parasitoid wasps. This resource provides a foundation for investigating the functional and evolutionary significance of new genes in driving phenotypic innovation within parasitoid wasps.

### 3.2. The Structural and Evolutionary Analysis of the New Genes

**Figure 2:**
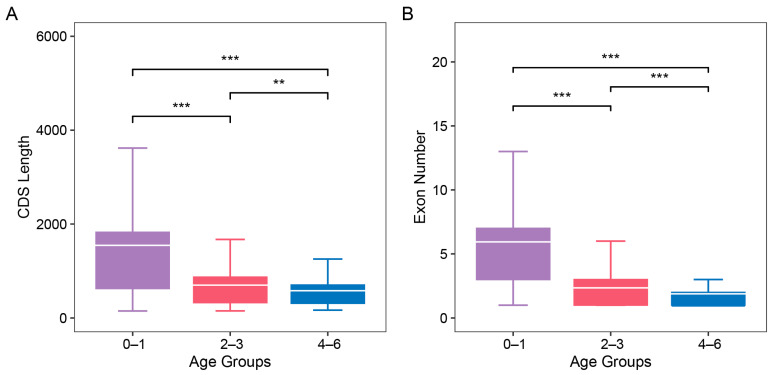
The CDS length (**A**) and exon numbers (**B**) of three age groups (branch 0–1, branch 2–3, and branch 4–6). The white bar indicates the average of each age group. Wilcoxon’s test was used to calculate significance between age groups. *p*-value between age groups was calculated by Wilcoxon’s test, with significance levels indicated as follows: ** *p* < 0.01; *** *p* < 0.001.

Studies across diverse lineages, including *Drosophila*, plants, and mammals, have shown that new genes are typically short in sequence and predominantly consist of single exon [5,16]. We conducted a comparative analysis of coding sequence (CDS) length by categorizing genes into three age groups: branch 0–1 (oldest genes), branch 2–3 (middle-aged genes), and branch 4–6 (new genes). The oldest genes in branch 0–1 showed a significantly longer CDS length comparing to the middle-aged genes and the new genes (one-sided, unpaired Wilcoxon test, *p* < 0.05). Similarly, the middle-aged genes had significantly longer CDS lengths than the new genes (one-sided, unpaired Wilcoxon test, *p* < 0.05) (Figure 2A).

We also compared the number of exons among the three age groups. The oldest genes had significantly more exons than both the middle-aged and new genes (one-sided, unpaired Wilcoxon test, *p* < 0.05). And the middle-aged genes also had more exons than the new genes, with this difference being statistically significant (one-sided, unpaired Wilcoxon test, *p* < 0.05) (Figure 2B). Among the 480 new genes, 280 were single-exon genes, 155 (55.36%) of which originated from RNA-mediated duplication or de novo processes. This aligns with expectations for RNA-mediated duplicates, which are commonly single-exonic. However, a number of new genes (45.9%) originating from DNA duplication are also single-exonic, which warrants further investigation.

After gene duplication, newly duplicated genes might be an immediate source of functional novelty under the selective pressure to survive genetic erosion [48]. Our dating results provide a framework for identifying new duplicate genes (child) and their closest old paralogs (parent), enabling us to explore whether new duplicates evolve under distinct evolutionary pressures compared to their parental copies. Since protein functional divergence follows from the ratio of Ka/Ks non-synonymous substitutions, we used PAML package to perform paralogous Ka/Ks test applied in 94 parent–child gene pairs [49]. After filtering genes with outlier values (see details in methods), 19 out of 94 pairs of tested parent–child copies (20.21%) were significantly lower than 0.5, indicating functional constraint on both parent–child genes (Ka/Ks < 0.5, *p* < 0.05) (Supplementary Table S4). In contrast, 249 out of 878 pairs (29.1%) of old duplicate genes in branch 0-3 were under negative selection (Ka/Ks < 0.5, *p* < 0.05), which reflects those old duplicate genes generally undergo stronger selective constraints (Supplementary Table S5). The proportion of old genes under negative selection was significantly higher than the new duplicate genes. The precious studies in primates and flatfishes also showed the same results, whereas the proportion of new duplicate genes under negative selection in our study is positioned between that of primates and flatfishes [6,7]. This indicates new duplicates experienced a unique selection pressure within parasitoid wasps.

### 3.3. Tissue-Specific Expression Pattern of New Genes

**Figure 3:**
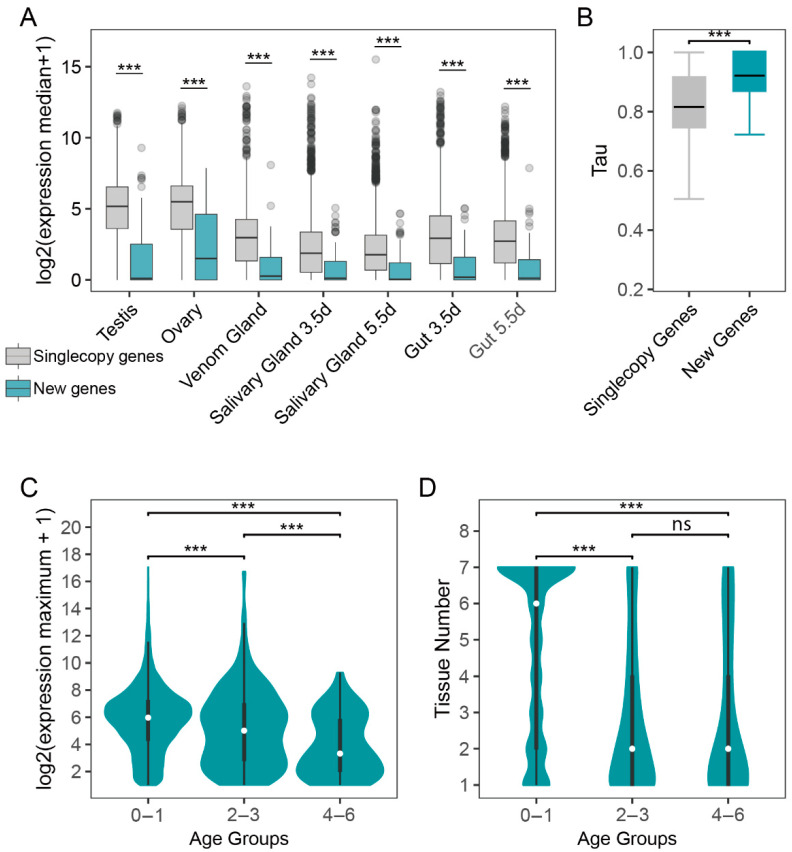
Expression patterns of the new genes. (**A**) The log-scale expression of new gene and single copy genes for different tissues. The relative gene expression was calculated using log2 (median TPM + 1). (**B**) The tissue-specific score between new genes and old singleton genes. The black bar indicates the average. (**C**,**D**) Log2-based maximum expression levels across seven tissues and the number of tissues where genes were expressed (TPM > 1). Interquartile ranges are represented by black bars, while the violin curve illustrates the probability density of the data, with the median value indicated by a white dot. Genes were categorized into three groups based on their age identified in Figure 1A. *p*-value between age groups was calculated by Wilcoxon’s test, with significance levels indicated as follows: ns: not significant. *** *p* < 0.001.

Comparative multi-transcriptome analyses of *Drosophila* and mammalian adult tissues have suggested that new genes tend to be testis-specific, whereas older genes are more commonly ubiquitously expressed or exhibit specificity in somatic tissues [50,51,52]. This observation led to the proposal of the “out of the testis” hypothesis, which posits that new genes initially gain functionality in the testis, possibly due to its permissive transcriptional regulation. This implied that new genes acquire new functions by going through unique expression alterations. To explore the expression pattern of the new genes in *P. puparum*, we calculated the expression level (TPM) of all annotated genes across seven RNA-Seq datasets, including samples from yellow-pupae testis, adult ovary, adult venom gland, adult gut, and larval salivary gland. We found that new genes had significantly lower expression levels in all the tissues compared to single-copy older genes, which are defined as one-to-one orthologs across seven parasitoid species (Figure 3A). To ensure reliable tissue-specific analysis, we filtered out genes with TPM < 1 across all tissues, leaving 72 new genes for which tissue-specific tau (*τ*) scores were calculated. Tau scores range from 0 (ubiquitous expression) to 1 (strong tissue specificity) (Supplementary Table S6). The tissue specificity of 72 new genes was significantly higher than that of the 3544 single-copy older genes conserved in the seven species (one-sided, unpaired Wilcoxon test, *p* < 0.05) (Figure 3B). Among 56 of 72 new genes exhibited tissue-bias expression, 12 of the 56 new genes (21.4%) exhibited testis-specific expression (*τ* > 0.85), with 6 originating from DNA-mediated duplication and more than half having TPM values below 10. In contrast, five of six de novo genes demonstrated higher testis-bias expression, with median TPM values exceeding 10 (Supplementary Table S6). Interestingly, a larger proportion of new genes exhibited tissue-specific expression biased toward the ovary (23 out of 56 new genes, 41.07%) or venom gland (15 out of 56 new genes, 26.79%). Among the 15 new genes with venom bias, 13 new genes (branch 4 to branch 6) originating from DNA-mediated and RNA-mediated duplication showed low expression levels. Therefore, we investigated the changes in expression patterns of new duplicates and their parental genes. Among 13 parent–child gene pairs with a median TPM greater than one in multiple tissues, six child genes exhibited a shift in tissue specificity compared to their respective parent genes. For instance, the child gene *SPOPL* in branch 4 (Speckle-type POZ protein-like) demonstrated a maximum expression in the venom gland compared to other tissues despite a tau score of less than 0.85 (TPM = 7.98). In contrast, the parent gene CON (Connectin) displayed a higher expression level (TPM > 100) in ovary and testis.

Furthermore, both venom-bias de novo genes exhibited a low-to-high expression trend: a de novo gene in branch 4 with TPM > 100 and the other de novo gene in branch 6 with TPM < 10. Thus, we discovered that the gene expression patterns varied across different gene age groups by analyzing transcription profiles in seven tissues of *P. puparum*. Specifically, new genes were expressed in only one tissue, with a median TPM value of less than one, while the oldest gene groups (branch 0–1) exhibited expression across six tissues, with a median TPM value exceeding 5 (Figure 3C,D). When comparing expression abundance and breadth, the oldest genes showed significantly higher expression levels than both middle-aged and new genes (one-sided, unpaired Wilcoxon test, *p* < 0.05) (Figure 3C,D). The middle-aged genes also show significant differences from new genes in terms of expression levels and the number of expressed tissues (one-sided, unpaired Wilcoxon test, *p* > 0.05). This suggests the presence of an age-dependent expression trend in parasitoid wasps, a phenomenon also observed in previous studies on new genes in mammals, fish, and plants [5,7,45].

### 3.4. A New Gene Serves as a Hub Gene of Venom Gene Regulatory Network

**Figure 4:**
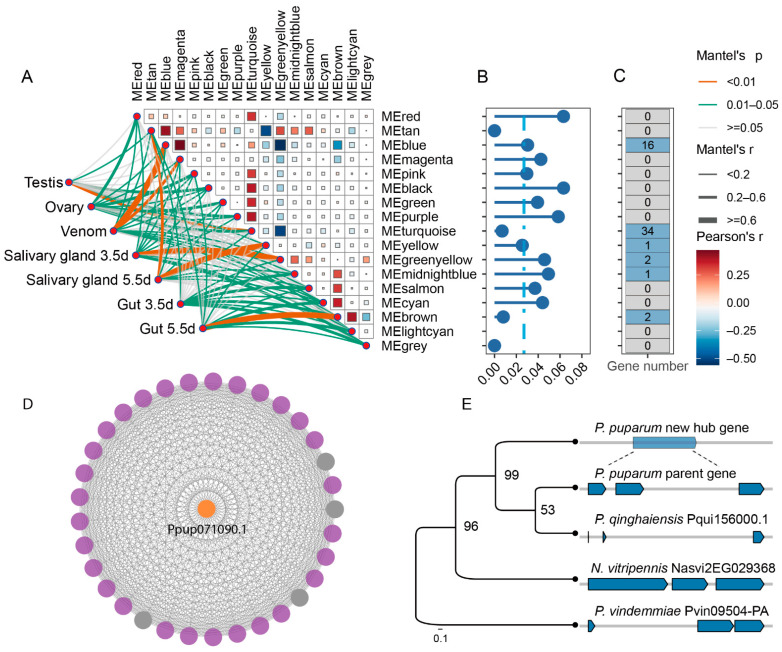
Expression network of new and old genes across the different tissues. (**A**) Gene weight co-expression network. (**B**) The percentage of the new genes in the different modules. The vertical blue line represents the genome-wide percentage of the new genes (2.71%), and the proportion of new genes are indicated by blue points. (**C**) Heatmap of the tissue-specific index of the new genes across different modules. (**D**) The venom-related network was visualized, highlighting a hub gene with the highest degree of connections and its associated interactions. In the plot, orange node represents the core new gene, purple nodes represent venom-biased genes, and grey nodes represent non-biased genes. (**E**) Exon–intron structure of parental genes (blue) and new hub (orange) gene.

Upon carefully examining the new genes we identified, alongside the 179 venom genes previously reported [41], we found no overlap between the two groups. This suggests that the new genes may not yet directly contribute to the innovation of the venom composition in parasitoid wasps. Therefore, we hypothesize that the new genes expressed in the venom gland may play a regulatory role associated with venom gene expression. To test this hypothesis, we utilized seven RNA-Seq datasets from the studied tissues (testis, ovary, venom gland, salivary gland, and gut) to construct a weighted gene co-expression network. This analysis clustered 14,622 genes into 17 modules based on expression similarity, with module sizes ranging from 53 to 9542 genes (Figure 4A). The turquoise module (MEturquoise), comprising 9542 genes, showed a strong association with the ovary and testis (0.6 ≤ R^2^ ≤ 0.8, *p* < 0.05). Remarkably, 59.72% (43/72) of 72 expressed new genes were identified within this module. Among these, 27.90% (12 genes) exhibited testis-biased expression, while 51.16% (22 genes) displayed ovary-biased expression (*τ* > 0.85) (Figure 4C). Of the 17 modules analyzed, only one module showed a strikingly strong correlation with the venom gland (R^2^ = 0.94, *p* < 0.05), identified as the blue module, which contains 1749 genes and is closely associated with the venom gene regulatory network (Figure 4A). Within this module, 334 of the 1749 genes exhibited venom-biased expression, indicating that older genes play a significant role in the venom co-expression network. The percentage of the new genes in the blue module was higher than the genome-wide percentage of the new genes (Figure 4B). Specifically, 19 new genes were presented in this venom-related gene network, with 15 exhibiting venom-specific expression, suggesting their potential roles in venom-related functions (Figure 4C). To evaluate the contribution of new genes to the venom-related network, we calculated module membership (kME) values, ranging from −1 to 1, to quantify each gene’s connectivity with other genes in the module. Genes with high kME values, known as hub genes, occupy central positions within the network. Based on their high connectivity, we identified a new gene (*Ppup071090.1*) as a hub gene, which originated from a three-exon parental gene through RNA-mediated duplication (Figure 4D,E). To validate the reliability of this new hub gene, we first conducted RT-PCR analysis, which confirmed the specifical presence of *Ppup071090.1* in ovarian and venom gland tissues (Supplementary Figure S2). Subsequent RT-qPCR quantification revealed the expression levels of *Ppup071090.1* were significantly higher in the ovary and venom gland compared to testis, larval salivary gland, and carcasses (without ovary and venom gland) (*p* < 0.05) (Supplementary Figure S3).

Further GO enrichment analysis of the genes in the hub-related gene network revealed their involvement in pathways such as protein N-linked glycosylation, nucleosome assembly, and nucleosome organization (Supplementary Figure S1). Notably, 41 venom genes (41/179, 23% of all venom genes) were identified in this network [41]. These findings indicate that the new gene has not been directly utilized as a venom protein-coding gene; instead, it may function as a central hub involved in the regulation of venom genes.

## 4. Discussion

New gene origination plays a major driving force for understanding evolutionary mechanisms underlying new traits and adaptive functions. An excellent example of this is the venom system of parasitoid wasps. As significant evolutionary innovations, venom offers an exceptional opportunity to investigate how new genes contribute to the phenotypic adaptations of these wasps to their diverse natural hosts. In this study, we analyzed a representative group of parasitoid wasps to explore the role of new genes, uncovering insights into their origination mechanisms and evolutionary roles. Our results show that gene duplication is the dominant origin of the new genes in parasitoid wasps, consistent with its role as a critical evolutionary process for generating genetic novelty [53]. Similar mechanisms have been observed in other taxa, such as *Drosophila*, mammals, and flatfishes [6,7,44]. Notably, the evolutionary origins of intron-free new genes derived from ancestrally intron-free parental genes remain a persistent challenge in the identification of retrogenes. Furthermore, more than half of the child duplicates retain a similar expression pattern to their parent paralogous genes. This supports the existence of “responsive backup circuits” across various species, where a redundant gene copy is upregulated when its paralog is subjected to an inactivating perturbation [46].

Combining gene age analysis with expression patterns, we identified the testis as a hotspot for new gene origination, consistent with its high transcriptional activity and permissive chromatin state [54,55]. However, our results reveal that new genes in parasitoid wasps are not solely restricted to the testis. Many new genes also expressed in venom gland, emphasizing the role of venom systems as evolutionary innovations that recruit new genes over short evolutionary time scales. A plausible explanation for this rapid turnover in venom-related genes lies in the diverse parasitism strategies of parasitoid wasps, which require continuous updates to their venom repertoire to adapt to a variety of hosts [20,41]. Although no new genes were found to have become venom proteins in *P. puparum*, several acquired the venom expression and were integrated as hub genes within existing venom-related gene networks. These hub genes participated in metabolic pathways and interacted extensively with older genes. Interestingly, older genes predominated within the venom-related co-expression networks, suggesting that they play a more pervasive role in maintaining the stable regulation of venom functions. A similar regulatory mechanism has been observed in human-specific gene networks associated with brain development [56,57]. For new genes to acquire functional relevance, they often become incorporated into pre-existing genetic interaction networks. This integration enables them to gain associated biological activities. New genes, with their shorter coding sequences, often feature intrinsically disordered regions and low-complexity sequences, characteristics that increase their binding flexibility and adaptability [58,59]. These advantageous properties facilitate their rapid incorporation into gene networks as hubs, where they interact with a wide range of partners.

In summary, we identified new genes across a group of representative parasitoid wasps, shedding light on their roles in the rapid evolution of venom systems. Our findings emphasize the importance of new genes in driving venom evolution and highlight the molecular mechanisms behind their emergence and integration. These results provide valuable insights into the evolutionary dynamics of new gene functions and represent a significant contribution to our understanding of venom evolution in parasitoid wasps.

## Data Availability

Raw data are provided in spreadsheets and can be downloaded at Supplementary Materials.

## Supplementary Materials

The following supporting information can be downloaded at https://www.mdpi.com/article/10.3390/insects16050502/s1. Supplementary Table S1: List of species and data resources used in this study. Supplementary Table S2: BUSCO scores of the seven species. Supplementary Table S3: List of new genes. Supplementary Table S4: Duplicated new genes under natural selection. Supplementary Table S5: Duplicated older genes under natural selection. Supplementary Table S6: Seventy-two new genes expressed in more than one tissue with median TPM > 1. Supplementary Table S7: The primers for qRT-PCR. Supplementary Figure S1: GO enrichment analysis of the genes in the hub-related gene network. Supplementary Figure S2: Validation of new hub gene *Ppup071090.1* expression pattern through RT-PCR analysis in various tissues. Supplementary Figure S3: Relative expression levels of new hub gene *Ppup071090.1* across various tissues.
