# Supplementary material for: Genomic Analysis Reveals the Role of New Genes in Venom Regulatory Network of Parasitoid Wasps
Source: Insects. 2025 May 7;16(5):502. doi: 10.3390/insects16050502 (PMC12112512; doi:10.3390/insects16050502)
Supplement: Supplementary file 1 [file insects-16-00502-s001.zip › Supplementary Figures.pdf]

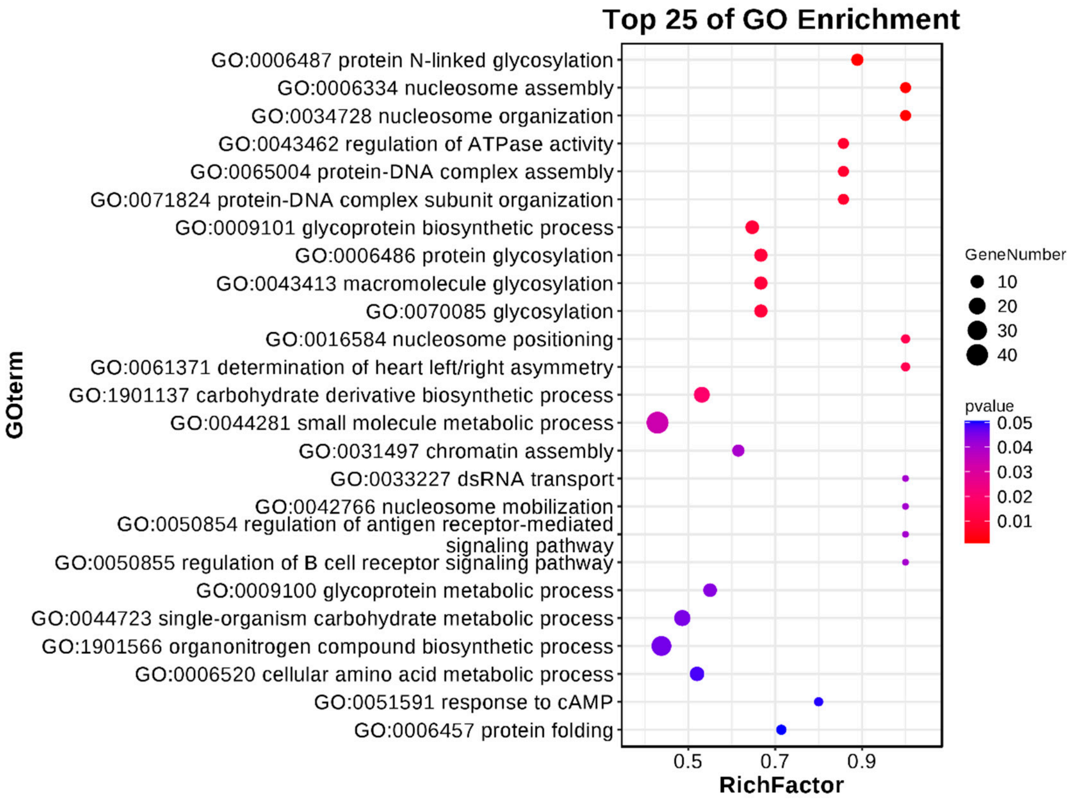

Supplementary Figure S1 GO enrichment analysis of the genes in the hub-related gene network

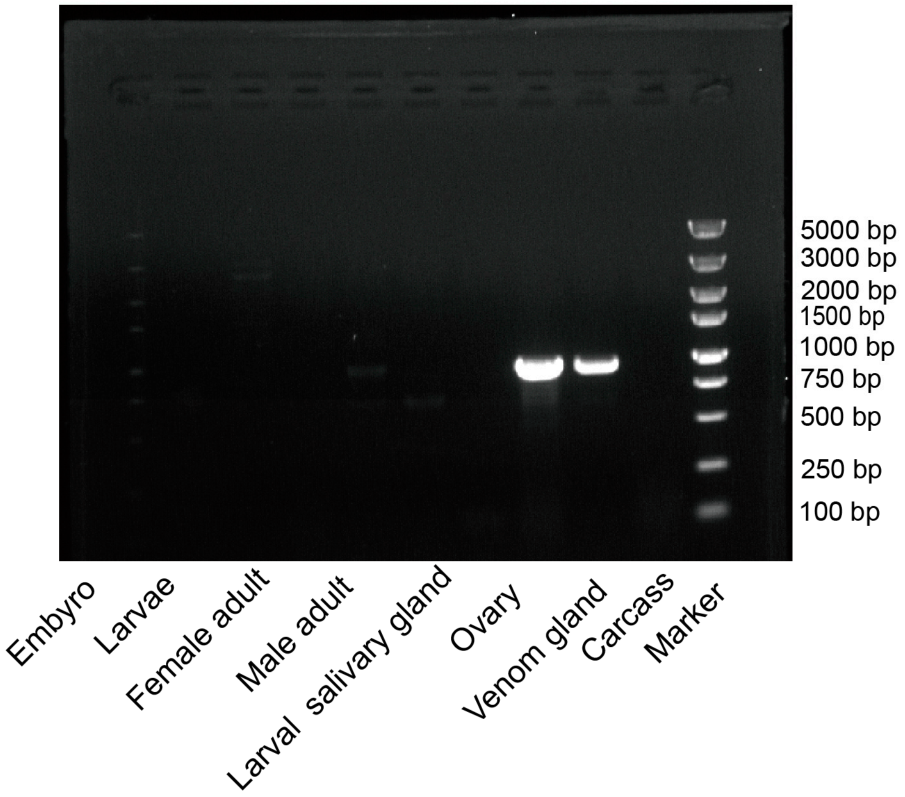

Supplementary Figure S2 Validation of new hub gene *Ppup071090.1* expression pattern

through RT-PCR analysis in various tissues

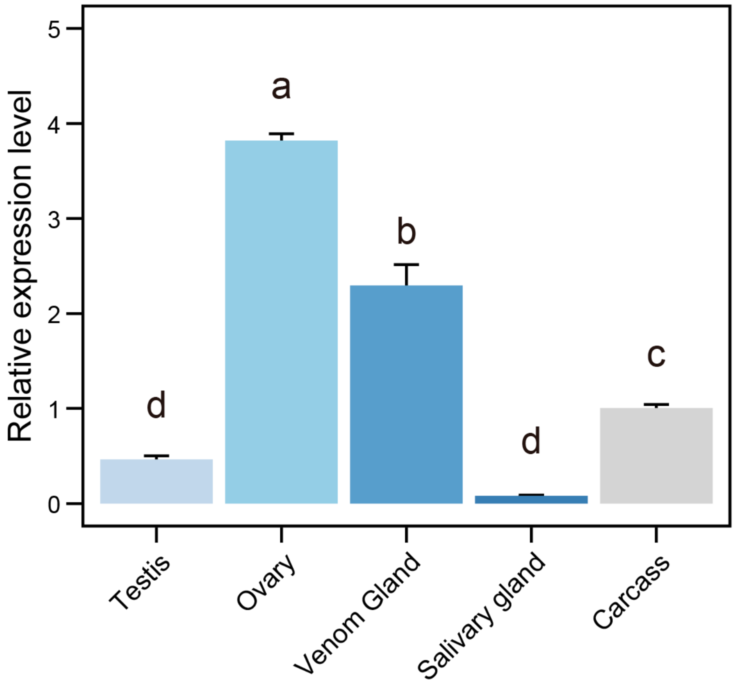

Supplementary Figure S3 Relative expression level of new hub gene *Ppup071090.1* in different tissues. n = 3 biological replicates. Expression values are mean ± standard error of the mean. Lowercase letters indicate significant differences between tissues using ANOVA and Tukey's HSD test for multiple comparisons ($P < 0.05$).
